# Visits to Private Asylums

**Published:** 1903-10-03

**Authors:** 


### VISITS TO PRIVATE ASYLUMS.

By OUR RAMBLING REPORTER.

### THE SILVER BIRCHES, EPSOM.

THIS asylum stands in the old town of Epsom, and is within a few minutes' walk of the station. The house dates back to the time of Henry VIII., but of course it has been modernised to suit the requirements of later generations, although it still retains many of the characteristics of more ancient days. It is licensed for the reception of 14 lady patients, and the license is 60 years old. The grounds extend to six acres and a half. Much of this is laid out in lawns and orchards, but some has been left as a meadow for purposes of recreation. Certainly the gardens are most beautiful and have a delightful old-world flavour about them. They are quite private and well sheltered from the colder winds, while one of the walks is so much overhung with thick foliage that it is cool in the hottest day of summer. The house itself is admirable. All the rooms are comfortably furnished, and everything is in good order. It is almost unnecessary to say that restraint and seclusion are not employed. It is a "home" and not an "asylum" in the common meaning of the word. Dr. Clement Daniel is the proprietor and resident medical officer. Altogether the Silver Birches is a charming place for its purpose—a sort of haven of rest for the mental invalid—and it deserves to be better known to medical practitioners who may have patients to certify, and often hardly know where to send them, and also to solicitors who may have to make arrangements for the care of the insane. Epsom is, of course, easily reached from London and is in direct communication with many parts of Surrey and Sussex.

